# The study of the association between immune monitoring and pneumonia in kidney transplant recipients through machine learning models

**DOI:** 10.1186/s12967-020-02542-2

**Published:** 2020-09-29

**Authors:** Bo Peng, Hang Gong, Han Tian, Quan Zhuang, Junhui Li, Ke Cheng, Yingzi Ming

**Affiliations:** 1 Transplantation Center, The Third Xiangya Hospital, Central South University, No. 138 Tongzipo Road, Changsha, Hunan 410013 P. R. China; 2 SING Lab, The Hong Kong University of Science and Technology, Hong Kong, P. R. China

**Keywords:** Immune monitoring, Kidney transplant, Machine learning, Pneumonia, Immunosuppression

## Abstract

**Background:**

Kidney transplantation is the optimal treatment to cure the patients with end-stage renal disease (ESRD). However, the infectious complication, especially pneumonia, is the main cause of mortality in the early stage. Immune monitoring by relevant biomarkers provides direct evidence of immune status. We aimed to study the association between immune monitoring and pneumonia in kidney transplant patients through machine learning models.

**Methods:**

A total of 146 patients receiving the immune monitoring panel in our center, including 46 pneumonia recipients and 100 stable recipients, were retrospectively reviewed to develop the models. All the models were validated by external data containing 10 pneumonia recipients and 32 stable recipients. The immune monitoring panel consisted of the percentages and absolute cell counts of CD3^+^CD4^+^ T cells, CD3^+^CD8^+^ T cells, CD19^+^ B cells and natural killer (NK) cells, and median fluorescence intensity (MFI) of human leukocyte antigen (HLA)-DR on monocytes and CD64 on neutrophils. The machine learning models including support vector machine (SVM), logistic regression (LR), multi-layer perceptron (MLP) and random forest (RF) were applied for analysis.

**Results:**

The pneumonia and stable groups showed significant difference in cell counts of each subpopulation and MFI of monocyte HLA-DR and neutrophil CD64. The SVM model by monocyte HLA-DR (MFI), neutrophil CD64 (MFI), CD8^+^ T cells (cells/μl), NK cells (cell/μl) and TBNK (T cells, B cells and NK cells, cells/μl) had the best performance with the average area under the curve (AUC) of 0.940. The RF model best predicted the patients who would progress into severe pneumonia, with the average AUC of 0.760. All the models had good performance validated by external data.

**Conclusions:**

The immune monitoring panel was tightly associated with pneumonia in kidney transplant recipients. The models developed by machine learning techniques identified patients at risk and predicted the prognosis. Based on the results of immune monitoring, better individualized therapy might be achieved.

## Background

Kidney transplantation is the optimal treatment to cure the patients with end-stage renal disease (ESRD) [1]. Novel immunosuppressive drugs improve the prognosis of kidney transplantation and minimize the side effects, but infection, especially pneumonia, remains one of the main challenges in the early stage after kidney transplantation [2–4]. Over immunosuppression significantly impairs host immunity, leading to increased risk of infection. Currently, the routine surveillance in clinic is therapeutic drug monitoring [5, 6]. However, this strategy is quite restrictive and only provides indirect evidence of patient immune response. The high inter- and intra-patient variability and the deficiency of limited sampling strategies used in therapeutic drug monitoring increase the risk of graft failure [5, 7]. In contrast, the immune monitoring could provide direct information on patient response to immunosuppressive drugs or pathogens, thus contributing to better individualized therapy and long-term prognosis [8–10].

In recent years, a quantity of immune biomarkers have been found to diagnose or predict infection in solid organ transplant recipients, making them as parameters for immune monitoring. These include cell counts of lymphocytes, CD4^+^ T cells, CD8^+^ T cells and natural killer (NK) cells, CD4/CD8 ratio, molecule expression on specific cells such as human leukocyte antigen (HLA)-DR on monocytes, CD64 on neutrophils and programmed cell death protein 1 (PD-1) on lymphocytes, immunoglobulins, complements, soluble CD30 and immune cell response to stimuli [11–13]. However, in majority of these studies, parameters are usually analysed separately, or patients are simply classified by scoring according to the parameters. Single parameter without exact weight could not reflect the immune status accurately. Without comprehensive analysis by appropriate statistical methods, the efficacy of these parameters may not be ideal.

Compared with traditional methods, the machine learning techniques have advantages in big data processing. They have high power and accuracy, and can deal with numerous parameters simultaneously. Recently, these techniques have been used in therapeutic drug monitoring [14], and to predict the prognosis of chronic kidney disease [15]. Our group also reported the application of machine learning models to predict tacrolimus stable dose in kidney transplant recipients [16]. The application of machine learning techniques in immune monitoring is promising, which may help us better understand the complexity of immune system [17].

In this study, we retrospectively analysed the kidney transplant recipients who underwent the immune monitoring panel in our center, and developed machine learning models to study the association between the immune monitoring results and pneumonia in kidney transplant recipients.

## Methods

### Study design

This was a retrospective, case–control study to evaluate the association between the immune monitoring panel and pneumonia in kidney transplant recipients. Both inpatients and outpatients who underwent the test of the immune monitoring panel from November 1^st^, 2017 to December 31^st^, 2019 in the Transplantation Center, The Third Xiangya Hospital, Central South University were enrolled to develop the machine learning models. Subsequently, the models were validated by the external data containing patients who received the test from January 1^st^, 2020 to March 31st, 2020 in our center. The exclusion criteria included (1) age less than 18 years old or more than 65 years old; (2) non-solid organ transplant patients or other transplant recipients; (3) multiple transplants; (4) less than or equal to 3 months post kidney transplant; (5) rejection, tumor or other infection; (6) rituximab administration. All patients who were not excluded were enrolled. The study was approved by the Institutional Review Board of Third Xiangya Hospital, Central South University (No. 2019-S448).

### Patients

All the kidney transplant recipients received the allografts from donation after citizen’s death (DCD) or from close family members after 2012. All the transplants performed in our center were approved by the DCD Ethics Committee of the Third Xiangya Hospital, Central South University. The allograft was attributed by the China Organ Transplant Response System. Anti-thymocyte globulin (ATG) (1.00 mg/kg daily for 3 days) or basiliximab (20 mg at day 0 and 4) was used for induction treatment, and the standard triple immunosuppressive regimen including calcineurin inhibitor (CNI), mycophenolate mofetil (MMF) and corticosteroid was given as maintenance regimen. Pneumonia was diagnosed based on clinical symptoms, positive laboratory test results and significant imaging findings. Severe pneumonia was defined according to the previous publication (Additional file 1: Table S1) [18]. Because kidney transplant recipients had worse renal function compared with general population, one minor criterion of which blood urea nitrogen (BUN) was more than or equal to 20 mg/dL was increased to 40 mg/dL instead. Patients who met more than or equal to one major criterion, or more than or equal to three minor criteria were classified into severe pneumonia.

### Timing for receiving the immune monitoring test

All the patients received the immune monitoring test after kidney transplantation. For stable patients, they received the test at regular follow-up. Usually, they had the test every 3 to 6 months during the first year after transplantation, and then might extend to once a year.

For pneumonia patients, they received the immune monitoring test when diagnosed with pneumonia. The test was performed as a clinical routine when the pneumonia patient was admitted to hospital. Some patients received several tests, and only the result of the first test was recorded.

### Immune monitoring panel

The immune monitoring test consisted of two panels. One panel was BD Multitest 6-color TBNK reagent with BD Trucount tubes, which identified the percentages and absolute counts of CD3^+^CD4^+^ T cells, CD3^+^CD8^+^ T cells, CD19^+^ B cells and NK cells. This panel was performed according to the manufacture’s instruction and analysed by BD FACSCanto clinical software (BD Biosciences, San Jose, CA, USA). Another panel detected the median fluorescence intensity (MFI) of HLA-DR on monocytes and CD64 on neutrophils. It consisted of the following fluorochrome-conjugated monoclonal antibodies: anti-CD45-PerCP (peridinin-chlorophyll-protein, clone HI30, Biolend), anti-CD14-APC-Cy7 (allophycocyanin and cyanine dye 7, clone HCD14, Biolend), anti-HLA-DR-APC (allophycocyanin, clone L243, BD Biosciences) and anti-CD64-PE (phycoerythrin, clone 10.1, BD Biosciences). Briefly, 50 μl whole blood from the identical EDTA anticoagulation tube was used for detection. After erythrolysis, cells and monoclonal antibodies were incubated in dark for 15 min. After washing and resuspending, samples were detected using BD FACSDiva software. Both panels were performed using BD FACSCanto II.

### Model building

Four machine learning models including support vector machine (SVM), logistic regression (LR), multi-layer perceptron (MLP) and random forest (RF) were applied in this research. SVM, LR, MLP and RF were applied to study the association between immune monitoring panel and pneumonia in kidney transplant recipients. We adopted k-fold cross validation (k = 5) to find the optimal hyperparameters, and to estimate and compare the performance of different machine learning models. Generally, eligible patients were randomly and averagely divided into five subgroups. Four subgroups were used as the “derivation cohort” to develop the algorithm, and the remaining subgroup was used as the “validation cohort” to test the performance. After five rounds of training/validation rotation, the average sensitivity, specificity, positive predictive value (PPV), negative predictive value (NPV) and area under the curve (AUC) were calculated. The final algorithms were derived from the whole data of patients for developing models. Subsequently, the algorithms were validated by the external data.

The function of SVM model was $f$(x) = SIGN (β_0_ + β_m_x_m_). When the result was 1, it was classified into the pneumonia group; when the result was -1, it was classified into the stable group. The function of LR model was $f$(x) = SIGMOID (β_0_ + β_m_x_m_). When the result was more than 0.5, it was classified into the pneumonia group; when the result was less than or equal to 0.5, it was classified into the stable group. As one of most common artificial neural networks widely used in machine learning tasks, the MLP model could be regarded as a logistic regression classifier with transformed features through several non-linear neural network layers. The RF model adopted ensemble learning technique by using multiple decision tree classifiers together to predict the result. A total of ten trees were developed. Each tree was presented with a different part of the dataset for training. The final prediction result was obtained through majority voting.

These machine learning models were also applied to predict the prognosis of pneumonia. Pneumonia patients were classified into mild group and severe group. Similar strategy was adopted as described above. After five rounds of training/validation rotation, the average sensitivity, specificity, PPV, NPV and AUC were calculated.

All the machine learning models were built using the programming language Python 3.6 and its machine learning library scikit-learn.

### Statistical analysis

Continuous data were presented as the mean ± standard deviation (SD), and were compared using Student’s t-test, Welch’s t-test or the Mann–Whitney U test, where appropriate. Categorical data were compared using Pearson’s chi-squared (χ^2^) test or Fisher’s exact test, where appropriate. The performance of the models was assessed by calculating the area under the curve (AUC) of the receiver operating characteristic (ROC) curve. Statistical analysis was performed using SPSS version 22.0 (SPSS, Inc., Chicago, IL, USA). A *P*-value of < 0.05 was considered to be statistically significant.

## Results

### Basic characteristics

**Fig. 1:**
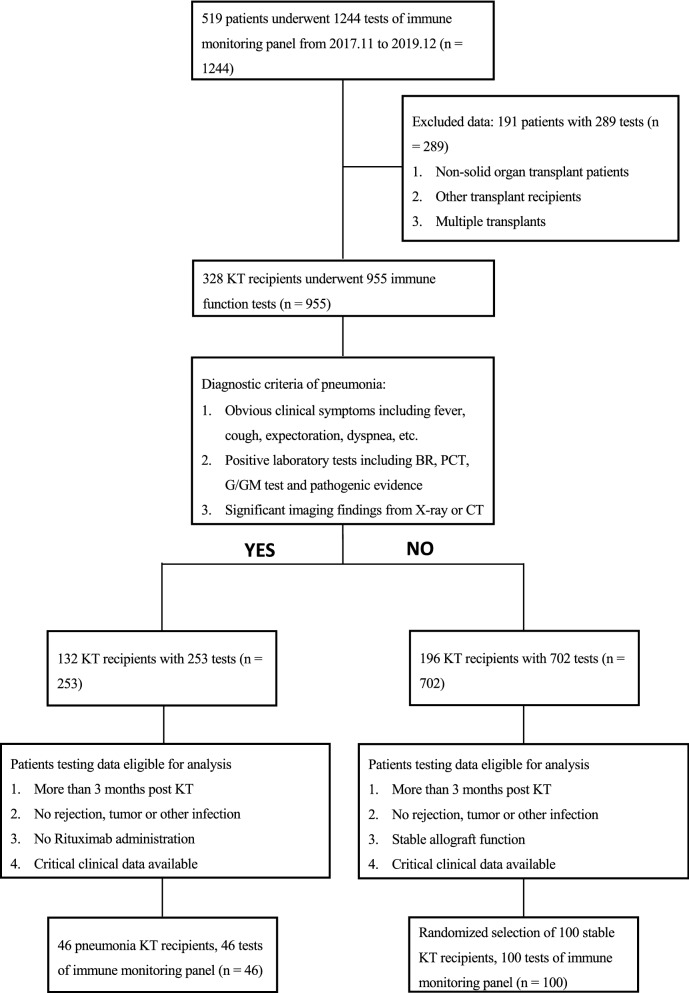
The study flowchart and exclusion criteria. 46 pneumonia and 100 stable kidney transplant recipients were finally enrolled for analysis. *KT* kidney transplant, *BR* blood routine, *PCT* procalcitonin, *CT* computed tomography

A total of 328 kidney transplant recipients underwent 955 tests of the immune monitoring panel from November 1st, 2017 to December 31st, 2019 in our center. A sizable part of the them were perioperative patients. Because the induction treatment had a significant impact on lymphocytes, only the patients more than or equal to three months post kidney transplantation were enrolled. The study flow was shown in Fig. 1.

46 eligible pneumonia patients underwent the immune monitoring test during the first week after admission to hospital. The average time from admission to test was 2.20 ± 2.08 days (2 patients received the test 7 days after admission and most cases received the first test within 3 days). Because they received the test when diagnosed with pneumonia, the average time from transplantation to receiving the test was the same as the time from transplantation to developing pneumonia, namely 14.67 ± 15.24 months. Among them, 29 cases (63.0%) were between 3 and 12 months post transplantation, and 17 (37.0%) cases developed into severe pneumonia. As control, 100 eligible stable kidney transplant recipients with the data of 100 tests were randomly selected. The average time from transplantation to receiving the test was 10.33 ± 8.47 months.

The clinical characteristics of the pneumonia group and the stable group showed no significant difference in age, gender, donor source, time since transplant and CNI regimen. Obviously, the stable group had a better allograft function than the pneumonia group. Because 16 patients in the pneumonia group received transplants in other hospitals, the induction treatment was not available. As a result, the induction treatment showed significant difference in these two groups. The details were shown in Table 1.

**Table 1:** Clinical characteristics of the patients

Characteristics	All (n = 146)	Pneumonia (n = 46)	Stable (n = 100)	P value
Age, years ± SD	40.61 ± 10.04	41.52 ± 8.01	40.19 ± 10.57	0.458
Male, n (%)	83 (56.8)	27 (58.7)	56 (56.0)	0.760
Donor, n (%)				0.098^*^
DCD	144 (98.6)	44 (95.7)	100 (100)	
Relative	2 (1.4)	2 (4.3)	0 (0)	
Time since transplant (months)	11.67 ± 11.15	14.67 ± 15.24	10.33 ± 8.47	0.732^#^
Induction, n (%)				< 0.001^*^
None	17 (11.6)	5 (10.9)	12 (12.0)	
ATG	106 (72.6)	22 (47.8)	84 (84.0)	
Basiliximab	7 (4.8)	3 (6.5)	4 (4.0)	
NA	16 (11.0)	16 (34.8)	0 (0)	
eGFR (ml/min/1.73 m^2^)	71.31 ± 23.93	59.11 ± 24.62	76.92 ± 21.50	< 0.001
CNI, n (%)				0.742^*^
FK506	135 (92.5)	42 (91.3)	93 (93.0)	
CsA	11 (7.5)	4 (8.7)	7 (7.0)	

Estimated glomerular filtration rate (eGFR) calculated by the Chronic Kidney Disease Epidemiology Collaboration (CKD-EPI) equation

*SD* standard deviation, *DCD* donation after citizens' death, *ATG* anti-thymocyte globulin, *NA* not available, *CNI* calcineurin inhibitor, *CsA* cyclosporine A

^*^ Tested by Fisher’s exact test; ^#^ Tested by Mann–Whitney U test

Similarly, patients receiving the immune monitoring test from January 1^st^, 2020 to March 31st, 2020 were collected as external data for validation. 110 patients received 174 tests, but after exclusion, 10 pneumonia patients and 32 stable patients were enrolled. The characteristics of these patients were shown in Additional file 1: Table S2.

### Immune status characterized by the panel

Compared with the stable group, the pneumonia group showed poor immune status, which was characterized by significantly lower cell counts of total T cells (CD3^+^ T cells), T cell subsets (CD4^+^ T cells and CD8^+^ T cells), B cells and NK cells (Table 2). Although the percentages of total T cells and NK cells showed statistical difference, they were not clinically significant (pneumonia vs stable, 76.79 ± 11.71vs 73.35 ± 10.28 for total T cells, *P* = 0.015; 12.78 ± 8.81 vs 17.11 ± 9.68 for NK cells, *P* = 0.003). The percentages of T cell subsets and B cells showed no significant difference. Notably, the CD4/CD8 ratio, which was reported as an immune biomarker, also showed no significant difference (pneumonia vs stable, 1.21 ± 0.61 vs 1.12 ± 0.59, *P* = 0.320).

**Table 2:** Immune monitoring panel of pneumonia and stable kidney transplant recipients

Parameters	All (n = 146)	Pneumonia (n = 46)	Stable (n = 100)	P value
Monocyte HLA-DR, MFI ± SD	1247.17 ± 764.82	931.17 ± 671.15	1392.53 ± 764.37	< 0.001
Neutrophil CD64, MFI ± SD	254.89 ± 409.29	589.20 ± 605.44	101.11 ± 54.08	< 0.001
CD3^+^ T cells/TBNK, mean ± SD (%)	74.44 ± 10.83	76.79 ± 11.71	73.35 ± 10.28	0.015
CD3^+^ T cells, n ± SD (cells/μl)	1024.10 ± 596.64	628.51 ± 365.86	1206.07 ± 595.30	< 0.001
CD8^+^ T cells/TBNK, mean ± SD (%)	36.56 ± 10.70	36.34 ± 9.97	36.66 ± 11.06	0.903
CD8^+^ T cells, n ± SD (cells/μl)	506.37 ± 343.93	294.23 ± 173.57	603.95 ± 359.21	< 0.001
CD4^+^ T cells/TBNK, mean ± SD (%)	37.03 ± 10.46	39.65 ± 12.98	35.82 ± 8.90	0.127
CD4^+^ T cells, n ± SD (cells/μl)	506.35 ± 295.64	335.48 ± 221.42	584.96 ± 293.13	< 0.001
NK cells/TBNK, mean ± SD (%)	15.74 ± 9.60	12.78 ± 8.81	17.11 ± 9.68	0.003
NK cells, n ± SD (cells/μl)	218.43 ± 179.47	107.39 ± 96.47	269.50 ± 185.96	< 0.001
B cells/TBNK, mean ± SD (%)	8.80 ± 5.10	9.41 ± 6.84	8.52 ± 4.07	0.601
B cells, n ± SD (cells/μl)	117.27 ± 92.39	67.36 ± 45.47	140.23 ± 99.36	< 0.001
TBNK, n ± SD (cells/μl)	1371.39 ± 751.49	809.90 ± 443.86	1629.67 ± 723.69	< 0.001
CD4/CD8 ratio, mean ± SD	1.15 ± 0.60	1.21 ± 0.61	1.12 ± 0.59	0.320

Tested by Mann–Whitney U test

*HLA-DR* human leukocyte antigen-DR, *MFI* median fluorescence intensity, *SD* standard deviation, *TBNK* T, B and NK cells, *NK cells* natural killer cells

The remaining two parameters also provided meaningful information. The expression of HLA-DR on monocytes was significantly lower in the pneumonia group (931.17 ± 671.15 vs 1392.53 ± 764.37, *P* < 0.001), while the expression of CD64 on neutrophils were much higher in the pneumonia group (589.20 ± 605.44 vs 101.11 ± 54.08, *P* < 0.001).

### Machine learning models based on immune monitoring

To study whether the parameters of the immune monitoring panel were associated with pneumonia in kidney transplant recipients, univariate LR was performed to assess each parameter (Additional file 1: Table S3). Several parameters including monocyte HLA-DR, neutrophil CD64 and cell counts of T cells, B cells and NK cells showed significance, but the performance was not ideal (data not shown).

**Fig. 2:**
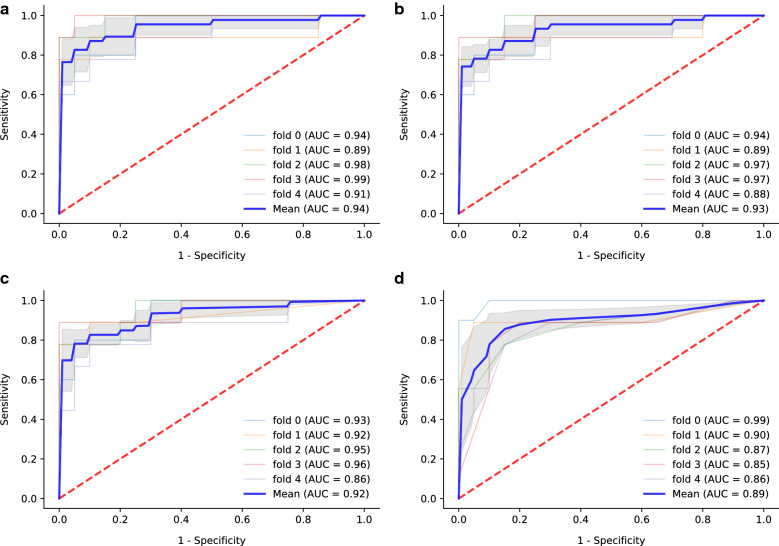
The ROC curves and average AUC of the machine learning models. K-fold cross validation (k = 5) was used to estimate and compare the performance of different machine learning models. After five rounds of training/validation rotation, the average AUC was calculated. **a** The support vector machine (SVM) model. **b** The logistic regression (LR) model. **c** The multi-layer perceptron (MLP) model. **d** The random forest (RF) model. ROC curve, receiver operating characteristic curve. *AUC* area under the curve

**Fig. 3:**
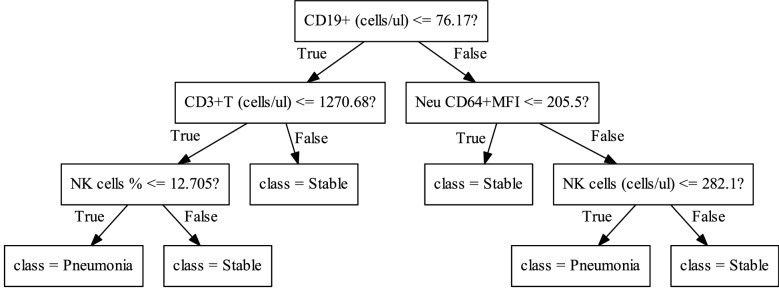
A one-tree example of random forest (RF) model. A total of ten trees were developed and one of them was shown in the figure. The final result was obtained through majority voting from ten trees

To improve the performance, machine learning models including SVM, LR, MLP and RF were developed as described in the methods. After five rounds of training/validation rotation, the average sensitivity, specificity, PPV, NPV and AUC of these modes were shown in Table 3. All the models had good results with AUC (Fig. 2), of which the SVM model had the highest AUC of 0.940. Notably, the SVM model also had good clinical practicality, with sensitivity of 81.7%, specificity of 92.0%, PPV of 83.6% and NPV of 91.3%. Monocyte HLA-DR (MFI), neutrophil CD64 (MFI), CD8^+^ T cells (cells/μl), NK cells (cell/μl) and TBNK (T cells, B cells and NK cells, cells/μl) were selected to build the SVM and LR models. The parameter coefficients were shown in Table 4. The MLP model, as one of the techniques of artificial neural network (ANN), calculated the probability of each category. The average AUC was 0.923, and the sensitivity, specificity, PPV and NPV were 71.8%, 92.0%, 82.7% and 87.9%, respectively. As an example, one tree of the RF model was shown in Fig. 3. A total of ten trees were developed. The final result was obtained through majority voting from the ten trees. The average AUC was 0.895, and the sensitivity, specificity, PPV and NPV were 73.6%, 95.0%, 88.0% and 89.2%, respectively.

**Table 3:** The performance of the models developed by machine learning to evaluate the risk of pneumonia

Models	Sensitivity (%)	Specificity (%)	PPV (%)	NPV (%)	AUC
SVM	81.7	92.0	83.6	91.3	0.940
LR	58.7	99.0	97.5	84.3	0.931
MLP	71.8	92.0	82.7	87.9	0.923
RF	73.6	95.0	88.0	89.2	0.895

*PPV* positive predictive value, *NPV* negative predictive value, *AUC* area under curve, *SVM* support vector machine, *LR* logistic regression, *MLP* multi-layer perceptron, *RF* random forest

**Table 4:** The coefficients of SVM and LR models

	Models	
Parameters	SVM	LR
Monocyte HLA-DR, MFI	− 0.000468	− 0.000386
Neutrophil CD64, MFI	0.00128	0.000852
CD8^+^ T cells, cells/μl	− 0.000512	− 0.000572
NK cells, cells/μl	− 0.00217	− 0.00201
TBNK, cells/μl	− 0.000398	− 0.000447
Constant	0.794	0.665

*SVM* support vector machine, *LR* logistic regression, *HLA-DR* human leukocyte antigen-DR, *MFI* median fluorescence intensity, *NK cells* natural killer cells, *TBNK* T, B and NK cells

Compared with mild pneumonia, severe pneumonia had a worse impact on allograft and patient survival. Among the 46 pneumonia patients, 17 cases progressed to severe pneumonia. Three patients died with functioning allografts, and one patient lost allograft. All of them were from the severe pneumonia group. Because all pneumonia patients received the immune monitoring tests early after admission (2.20 ± 2.08 days from admission to test), we also studied whether the result of the immune monitoring panel could predict the prognosis of pneumonia. The comparison between the two groups was shown in Table 5. Only the cell count of NK cells showed significance (135.60 ± 108.79 vs 59.28 ± 39.50, *P* = 0.027); the mild pneumonia group had higher monocyte HLA-DR, but not statistically significant (1068.59 ± 758.07 vs 696.76 ± 410.57, *P* = 0.127).

**Table 5:** The association of immune monitoring panel and prognosis of pneumonia in kidney transplant recipients

Parameters	Mild pneumonia (n = 29)	Severe pneumonia (n = 17)	P value
Monocyte HLA-DR, MFI ± SD	1068.59 ± 758.07	696.76 ± 410.57	0.127
Neutrophil CD64, MFI ± SD	584.17 ± 683.08	597.76 ± 462.88	0.657
CD3^+^ T cells/TBNK, mean ± SD (%)	75.42 ± 10.27	79.13 ± 13.85	0.065
CD3^+^ T cells, n ± SD (cells/μl)	657.84 ± 378.50	578.48 ± 348.62	0.453
CD8^+^ T cells/TBNK, mean ± SD (%)	34.31 ± 8.15	39.81 ± 11.96	0.070^*^
CD8^+^ T cells, n ± SD (cells/μl)	301.85 ± 169.62	281.23 ± 184.66	0.702^*^
CD4^+^ T cells/TBNK, mean ± SD (%)	40.42 ± 11.51	38.35 ± 15.46	0.635^#^
CD4^+^ T cells, n ± SD (cells/μl)	363.68 ± 231.05	287.37 ± 201.42	0.255
NK cells/TBNK, mean ± SD (%)	14.17 ± 8.55	10.41 ± 9.01	0.056
NK cells, n ± SD (cells/μl)	135.60 ± 108.79	59.28 ± 39.50	0.027
B cells/TBNK, mean ± SD (%)	9.21 ± 6.62	9.75 ± 7.40	0.946
B cells, n ± SD (cells/μl)	73.46 ± 48.64	56.96 ± 38.63	0.255
TBNK, n ± SD (cells/μl)	874.48 ± 470.51	699.75 ± 382.39	0.219
CD4/CD8 ratio, mean ± SD	1.28 ± 0.59	1.10 ± 0.64	0.323^*^
eGFR when discharge, ml/min/1.73 m^2^	78.27 ± 31.87	67.32 ± 35.23	0.285^*^
Death with functioning graft, n (%)	0 (0)	3 (17.65)	0.045^§^
Allograft loss, n (%)	0 (0)	1 (5.88)	0.370^§^

*HLA-DR* human leukocyte antigen-DR, *MFI* median fluorescence intensity, *SD* standard deviation, *NK cells* natural killer cells, *TBNK* T, B and NK cells

^*^Tested by Student's t-test. ^#^ Tested by Welch's t-test. ^§^ Tested by Fisher’s exact test. Others tested by Mann–Whitney U test. Estimated glomerular filtration rate (eGFR) calculated by the Chronic Kidney Disease Epidemiology Collaboration (CKD-EPI) equation

**Fig. 4:**
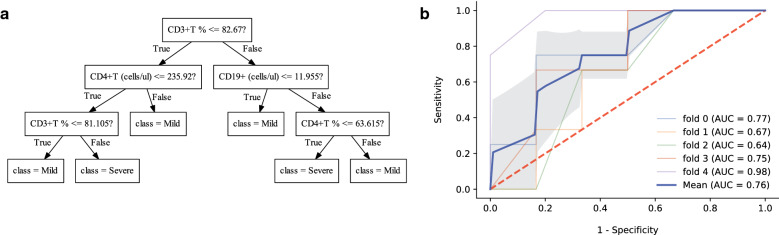
The random forest (RF) model to predict the prognosis of pneumonia in kidney transplant recipients. **a** A one-tree example of the ten trees. **b** The average AUC of RF model. *AUC* area under the curve

The machine learning models were used to predict the prognosis based on the immune monitoring panel, and the results were shown in Additional file 1: Table S4. The limited data had a negative impact on the effectiveness. The AUCs of SVM, LR, MLP and RF models were 0.600, 0.672, 0.716 and 0.760, respectively. Among them, the RF model had the best performance. A ten-tree RF model was developed, and one tree of the final algorithm was shown (Fig. 4a). Similarly, after five rounds of training/validation rotation, the average sensitivity, specificity, PPV and NPV of the RF model were 53.3%, 80.0%, 68.0% and 75.3%, respectively (ROC curve shown in Fig. 4b).

### Validation by external data

To further confirm the association between the immune monitoring test and pneumonia in kidney transplant recipients, the machine learning models were validated by the external data, which contained 10 pneumonia patients and 32 stable patients. All the models had good performance, with accuracy over 80%. The SVM model had the highest AUC of 0.945, and the sensitivity, specificity, PPV, NPV and accuracy were 90.0%, 81.3%, 60.0%, 96.3% and 83.3%, respectively. The details of other models were shown in Additional file 1: Table S5.

## Discussion

This study successfully provided a novel strategy to evaluate the significance of immune monitoring in kidney transplant recipients by machine learning models. Based on the results of immune monitoring panel, the SVM model best identified the kidney transplant recipients at risk of pneumonia, and the RF model best predicted the patients who would progress to severe pneumonia. All the models were validated by the external data, and showed good performance. The results of immune monitoring panel might contribute to better individualized therapy, including immunosuppressive drug adjustment and immunostimulant treatment.

Compared with traditional methods, the machine learning techniques could process multidimensional parameters simultaneously, and were not limited by data distribution [14]. Feature selection and parameter fitting were performed on training set, and evaluation of model performance was through validation set. Therefore, it had the ability of self-evolution by adjusting their structures when encountering errors [17]. The models could have better performance if more data obtained, making them promising in big data analysis. The immune system was exactly a very complicated network, and multiple parameters were needed to reflect the full picture of immune status. Therefore, the machine learning technique could be a powerful tool in analysis of immune monitoring.

The immune monitoring panel in this study consisted of the parameters that were relatively convenient and practical to obtain clinically. These included the percentages and absolute number of TBNK, HLA-DR on monocytes and CD64 on neutrophils. In our study, patients were not analysed by ATG and non-ATG groups due to the flaw of data from patients receiving transplantation out of our center. Because of the low ATG routine dose (1.00 mg/kg for 3 days) used in China, patients usually had lymphocyte reconstitution in three months (unpublished data). Therefore, we only enrolled patients more than three months post transplantation. Compared with the stable group, the pneumonia group had a much lower level of TBNK cell counts (including the respective subpopulations), lower expression of HLA-DR on monocytes but higher expression of CD64 on neutrophils. Fernández-Ruiz and colleagues reported that low TBNK cell counts in kidney transplant recipients predicted post-transplant opportunistic infection, and found that CD8^+^ T cells less than 100 cells/μl and CD4^+^ T cells less than 50 cells/μl at month 1 were the most valuable predictive parameters for non-ATG and ATG groups, respectively [2]. Luo and colleagues also reported lower cell counts and impaired function of CD4^+^ T cells, CD8^+^ T cells and NK cells in kidney transplant recipients with infection [19]. After analysis of machine learning models in our study, the cell counts of CD8^+^ T cells, NK cells and total TBNK were selected as parameters for SVM and LR model building, showing their importance in immune monitoring. The weight of these parameters was determined by the coefficients, which should be more accurate and reasonable than simple scoring [20, 21]. Notably, the percentages of TBNK subpopulations, including CD4/CD8 ratio, did not show significance. Similar result of CD4/CD8 ratio was observed in solid organ transplant patients, which showed poor diagnostic performance in infectious complications [22]. However, the inverted CD4/CD8 ratio (less than 1.0) was regarded as one of the parameters that defined immune risk phenotype in ESRD patients [21].

Monocyte HLA-DR and neutrophil CD64 were also important parameters in the models. Decreased expression of HLA-DR on monocytes was regarded as an unquestionable marker of monocyte anergy, which correlated with low cytokine release in response to bacterial challenges and reduced antigen presenting ability [11]. The clinical significance of monocyte HLA-DR has been verified in sepsis, and low monocyte HLA-DR expression was suggested as an indication for immunostimulant therapy [23]. In kidney transplant recipients, the expression of monocyte HLA-DR showed a significant decrease two weeks after transplantation compared with that before transplantation [24]. Monocyte HLA-DR also showed great difference between septic and non-septic groups after lung transplantation without modulating T cell reconstitution [25]. All these evidences proved that over immunosuppression not only impaired adaptive immunity, but also innate immunity.

The neutrophil CD64, on the contrary, remained low expression in the stable group. Once stimulated by inflammatory cytokines like interferon-γ (IFN-γ), a rapid and significant increase of expression of CD64 could be detected on neutrophils in 4 to 6 h, making it a sensitive indicator of systematic inflammation [26]. Importantly, immunosuppression treatment did not alter this characteristic. CD64 index even showed a better diagnostic performance of infectious complications than C reactive protein (CRP) or white blood cells in solid organ transplant patients [22]. Our study provided further evidence of clinical significance of neutrophil CD64 in kidney transplant recipients with pneumonia.

It must be noted that the algorithms derived from our study could not be utilized directly in other centers. It was because that the MFI of HLA-DR and CD64 in the models was relative value, which was determined not only by the expression intensity, but also by the setting of flow cytometer and the antibodies chosen. Only the MFI of fluorochrome PE, which had a fixed fluorochrome to antibody ratio, could be converted into absolute value of antibodies bound per cell (AB/C), making it possible for lab-to-lab standardization [11]. Another choice was using the internal reference microspheres, just like the commercial kit of Leuko64 (Trillium Diagnostics LCC, Meine, USA) to determine the CD64 index [22, 27]. Because this was not available in our center, MFI was directly used in the models. Nevertheless, the methodology to develop the models by machine learning could be adopted. Moreover, other valuable parameters, such as the concentration of immunoglobulins [28, 29] or complements [30, 31], could also be added to further improve the models [17].

Because this was a retrospective study, there were some limitations. The time point for the test of immune monitoring panel was not fixed, and the kinetic follow-up of immune monitoring was not available. The flaw of clinical data, including the definite etiological evidence, limited further stratification analysis. For machine learning techniques, the number of cases was relatively small. Further big data analysis or prospective cohort study was needed.

## Conclusions

This study established machine learning models to confirm the association between immune monitoring and pneumonia in kidney transplant patients. The SVM model consisting of monocyte HLA-DR (MFI), neutrophil CD64 (MFI), CD8^+^ T cells (cells/μl), NK cells (cell/μl) and TBNK (cells/μl) best identified patients at risk of pneumonia. The RF model predicted the prognosis of pneumonia. In the era of big data, comprehensive analysis based on multi-dimensional parameters was an effective method to deeply understand the complexity of diseases. The machine learning technique provided a good choice, which was promising in data analysis and contributed to better individualized therapy.

## Supplementary information

**Additional file 1:** Additional tables.

## Data Availability

The datasets used and/or analyzed during the current study are available from the corresponding author on reasonable request.

## Abbreviations

AB/C: Antibodies bound per cell
ANN: Artificial neural network
APC: Allophycocyanin
APC-Cy7: Allophycocyanin and cyanine dye 7
ATG: Anti-thymocyte globulin
AUC: Area under the curve
BUN: Blood urea nitrogen
CNI: Calcineurin inhibitor
CRP: C reactive protein
DCD: Donation after citizen’s death
ESRD: End-stage renal disease
HLA: Human leukocyte antigen
IFN-γ: Interferon-γ
LR: Logistic regression
MFI: Median fluorescence intensity
MLP: Multi-layer perceptron
MMF: Mycophenolate mofetil
NK: Natural killer
NPV: Negative predictive value
PD-1: Programmed cell death protein 1
PE: Phycoerythrin
PerCP: Peridinin-chlorophyll-protein
PPV: Positive predictive value
RF: Random forest
ROC: Receiver operating characteristic
SD: Standard deviation
SVM: Support vector machine
TBNK: T cells, B cells and NK cells
